# Magnetic Composite Scaffolds for Potential Applications in Radiochemotherapy of Malignant Bone Tumors

**DOI:** 10.3390/medicina55050153

**Published:** 2019-05-17

**Authors:** Florina Daniela Cojocaru, Vera Balan, Ionel Marcel Popa, Anca Munteanu, Anisoara Anghelache, Liliana Verestiuc

**Affiliations:** 1 Department of Chemical Engineering, Faculty of Chemical Engineering and Environmental Protection, Gheorghe Asachi Technical University, 700050 Iasi, Romania; mipopa@tuiasi.ro; 2 Department of Biomedical Sciences, Faculty of Medical Bioengineering, Grigore T. Popa University of Medicine and Pharmacy, 700454 Iasi, Romania; balanvera@yahoo.com (V.B.); liliana.verestiuc@bioinginerie.ro (L.V.); 3 Department of Medical Oncology-Radiotherapy, Faculty of Medicine, Grigore T. Popa University of Medicine and Pharmacy, 700115 Iasi, Romania; anca.munteanu@umfiasi.ro; 4 Regional Institute of Oncology, Department of Radiotherapy, 700483 Iasi, Romania; nanisoara@yahoo.com

**Keywords:** composite scaffolds, magnetic nanoparticles, radiotherapy, chemotherapy, doxorubicin

## Abstract

*Background and objectives*: Cancer is the second leading cause of death globally, an alarming but expected increase. In comparison to other types of cancer, malignant bone tumors are unusual and their treatment is a real challenge. This paper’s main purpose is the study of the potential application of composite scaffolds based on biopolymers and calcium phosphates with the inclusion of magnetic nanoparticles in combination therapy for malignant bone tumors. *Materials and Methods:* The first step was to investigate if X-rays could modify the scaffolds’ properties. In vitro degradation of the scaffolds exposed to X-rays was analyzed, as well as their interaction with phosphate buffer solutions and cells. The second step was to load an anti-tumoral drug (doxorubicin) and to study in vitro drug release and its interaction with cells. The chemical structure of the scaffolds and their morphology were studied. *Results:* Analyses showed that X-ray irradiation did not influence the scaffolds’ features. Doxorubicin release was gradual and its interaction with cells showed cytotoxic effects on cells after 72 h of direct contact. *Conclusions*: The obtained scaffolds could be considered in further studies regarding combination therapy for malignant bone tumors.

## 1. Introduction

Worldwide, cancer is the second most common cause of death, even though several advancements have been made concerning prevention, early diagnosis, and treatment protocols. The International Agency for Research in Cancer has registered more than 12 million new cases of cancer in a year and is estimated to reach over 21 million in 2030 [1].

Compared to other malignancies, primary bone tumors are quite rare, accounting for only 0.2% of all neoplasms in the UK and USA [2]. This fact certainly limits the data collection of their relative frequency. Primary bone tumors are less likely to metastasize than carcinomas, melanoma, or hematologic malignancies, such as plasmacytoma [3]. Other non-neoplastic conditions, such as inflammatory processes, bone cysts, fibrous dysplasia, non-ossifying fibroma, and Paget’s disease of the bone, exceed the cases of primary bone tumors [4]. Malignant bone tumors can occur spontaneously, but a substantial number of them do arise in the context of a hereditary disorder [5]. The most frequent bone tumors are osteosarcoma (35%), chondrosarcoma (25%), Ewing’s sarcoma (16%), chordoma (8%), and malignant fibrous histiocytoma (5%) [6].

The treatment of malignant bone tumors represents a huge clinical challenge, because surgical resection and radiation therapy are incapable of completely removing multifocal lesions. At the same time, chemical drugs may induce side effects and bone marrow microenvironment-associated drug resistance. Antiresorptive drugs like bisphosphonates and receptor activators of NF-κB antibodies are the current cure for bone metastases. However, their non-specific biodistribution is a significant problem [7]. Alternative therapies, such as hyperthermia, targeted therapy, immunotherapy or phototherapy, the use of nanoparticles, or stem cell transplants, have been proposed as potential alternatives [1].

Hyperthermia, used alone or in combination with other cancer therapies, is generally well tolerated and, if the temperature does not exceed 45 °C, rarely affects normal tissues. This is one of the main advantages over other treatment techniques [8]. Immunotherapy is based on the immune cells’ ability to recognize and target tumor cells, leading to their elimination. The advantages of this technique include high anti-tumor specificity and minimal side effects by utilizing the patient’s own immune system [9].

The treatment protocol of osteosarcoma (the most common bone tumor) includes surgical resection of the primary tumor and bone metastasis. The surgical margin reconstruction and adjuvant therapy plan are further delineated by the subtype of osteosarcoma. The bone defect is filled with bone graft material, to avoid fractures or bone deformations [10]. Due to the fact that tumoral tissue is very difficult to remove, surgical interventions are always followed by chemo/radiotherapy [11].

According to the National Cancer Institute, radiotherapy is defined as a cancer treatment that uses high doses of radiation to kill cancer cells and shrink tumors. At low doses, radiation is used in X-ray techniques to image the teeth or broken bones. At high doses, radiation kills cancer cells or slows their growth by damaging their DNA. Because radiation can be accurately limited to the depth of interest, radiotherapy is considered non-invasive and specific compared to other anti-tumor therapies. 

Concerning bone tumors, radiotherapy is thought to reduce, in the first stage, the bone mineral density shortly after radiation exposure, but subsequently induce re-calcification of the lesion in a process involved in bone remodeling. It is generally believed that, in patients responding to radiotherapy, about three months is needed for the bone to be sufficiently strengthened [12]. However, a potential side effect is the development of radio-resistant cells. Another problem is related to the excessively high radiation dose that can induce damage in adjacent normal tissues. For better therapeutic efficacy, several radio-sensitization strategies have been developed. Mostly, combinations of radiosensitizing chemotherapy and radiotherapy are used, providing better post-therapy outcomes [9].

Systemic chemotherapy is used in the treatment of cancer. Chemotherapeutic drugs or cytotoxic drugs are either synthetic or natural products or their derivatives, extracted from plants, marine species, and microorganisms [13]. Chemotherapy can have a number of limitations, such as toxicity and unfavorable biodistribution, generally being rapidly removed from the body. To reduce these problems, numerous targeting systems for the specific delivery of chemotherapeutics to tumor cells have been designed and evaluated [14].

In this context, polymers are appropriate materials to act as vehicles for encapsulated or chemically bonded drugs. Over time, polymers and polymeric nanoparticles have played a key role in the advancement of drug delivery technology providing controlled release of therapeutic agents in constant doses over long periods, cyclic dosage, and better efficiency of hydrophilic or hydrophobic drugs [15,16].

Three-dimensional porous scaffolds have been developed as an alternative method of treatment of common bone defects. These 3D scaffolds mimic the natural bone properties and should be biocompatible, osteoconductive, and should have adequate porosity and proper mechanical strength [17]. These scaffolds are intended to be used as bone graft materials to fill the void left behind after the surgical resection of the tumors. Magnetic nanoparticles (MNPs) were extensively studied for clinical diagnosis, magnetic resonance imaging, and carriers for targeted drug delivery [18]. Regarding the last application, MNP incorporation into 3D scaffolds was proposed as an alternative treatment for bone tumors [19]. It is essential for the MNPs to be superparamagnetic, meaning that they are magnetized only when they are exposed to a magnetic field [20]. 

Taking into account the concerns mentioned above, scaffolds based on biopolymers and calcium phosphates with inclusion of MNPs were prepared and characterized with the aim of proving their suitability in combination therapy (radiotherapy followed by chemotherapy) of malignant bone tumors. First, the influence of X-rays on the scaffolds was investigated, then an antitumor drug was loaded into the magnetic nanoparticles (before their inclusion in the scaffold). In vitro drug release and the interaction of the scaffolds with the bone cell line MG-63 were studied. 

## 2. Materials and Methods

### 2.1. Materials

The scaffolds were prepared using the following biopolymers: chitosan—Cs (Mw = 309.900 Da, degree of acetylation DA = 20.3; Vanson Chemicals, Redmond, WA, USA), hyaluronic acid sodium salt—Hya (solubility in H_2_O of 5 mg/mL, Sigma-Aldrich Chemie GmbH, Taufkirchen, Germany) and collagen − Col (type I + III, bovine origin, Lohman&Rauscher International, Rangsdorf, Germany). Magnetic nanoparticles (MNPs − magnetite coated with chitosan) were prepared using a protocol described by Balan et al. [21] and integrated in the mixture of the biopolymers. Calcium chloride (CaCl_2∙_2H_2_O) (M = 147.01 g/mol, solubility = 1280 g/L, Merck KGaA, Darmstadt, Germany), monosodium phosphate (NaH_2_PO_4∙_H_2_O) (pH = 4.1–4.5 at 25 °C, 5% in solution, M = 137.99 g/mol, Sigma Aldrich) and aqueous ammonia solution (NH_4_OH) (25% solution, Sigma Aldrich) were used in scaffold preparation during the co-precipitation process. As drug model, Doxorubicin hydrochloride (DOX) (M = 579.98 g/mol, Sigma Aldrich) was used. Lysozyme (HEWL, Fluka, Buchs, Switzerland) and collagenase *Clostridium histolyticum* (Sigma Aldrich) were used in biodegradation studies. Phosphate buffered solution (PBS), 0.01M pH = 7.2 was used for in vitro drug release experiments and to determine the PBS retention degree (%). Hank’s Balanced Salt Solution (HBSS), Dulbecco’s Modified Eagle’s Medium, high glucose with l-glutamate and pyruvate (DMEM), fetal bovine serum (FBS), Penicillin-Streptomycin-Neomycin (P/N/S), Thiazolyl Blue Tetrazolium Bromide (MTT) (all from Sigma Aldrich) were used for in vitro interactions studies of the magnetic scaffolds with cells. 

### 2.2. Preparation of the Magnetic Scaffolds

Six magnetic scaffolds were prepared using the protocol previously described in an article published in 2017 [22]. In brief, the scaffolds were obtained by co-precipitation of CaCl_2_ (40 wt%) and NaH_2_PO_4_ (25 wt%) [23] on a mixture of biopolymers solutions: Cs (1%), Col (1%) and Hya (1%) together with MNPs (5%), in the presence of NH_4_OH. The theoretical Ca/P ratio was 1.65. In Table 1 are detailed the magnetic scaffolds codifications, compositions, PBS retention degrees, magnetization and distinctive features.

### 2.3. X-ray Irradiation of the Scaffolds

Concerning radiotherapy, homogeneous irradiation with the prescribed dose of a target volume can be achieved. For the irradiation of the scaffolds, the first step was the CT (computed tomography) stimulation performed using CT-Sim Siemens Somatom Definition (Siemens, Erlangen, Germany). Due to the reduced thickness of the scaffold, it was placed on a 5 mm support, which was later included in the calculation of the irradiation parameters. After placing the metal parts, 1 mm CT sections of the sample were made. The irradiation of the material was performed with the Varian Clinac IX linear accelerator (Varian Medical Systems, Palo Alto, CA, USA). The steps were similar for the two irradiated scaffolds. 

**Figure 1:**
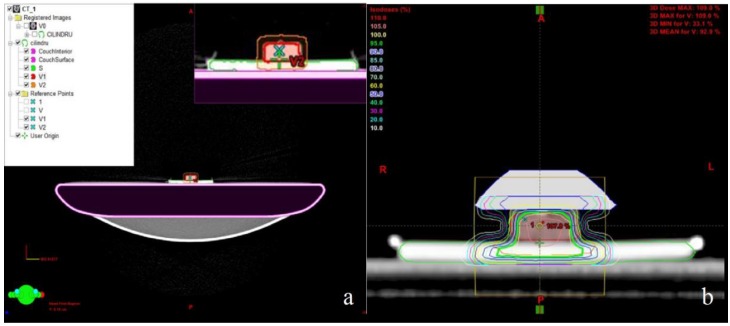
(**a**) The target volume delineation on the computed tomography (CT) images of the sample; (**b**) the dose distribution in volume of material after bolus application.

Within the irradiation plan called CILINDRU, the sections obtained were delineated—the external surface as the reference (S), the material (V1), and the target volume (V2), V2 = V1 + 5 mm—to compensate for possible position errors (Figure 1a). The treatment plan was done using the Software Planning: Eclipse™ Treatment Planning System (Varian Medical Systems). For a homogeneous dose distribution of the material volume, a bolus of 10 mm thickness was placed on the surface of the material, resulting in sufficient inclusion in the 95 isodose for V2 (95 isodose is shown in green in Figure 1b).

Characteristics of the used bolus have been previously described by our group [24]. Briefly, the bolus was used during the high energy photon and electron radiation treatments of different body areas, mostly the head, neck, chest wall, and vulva, in order to target the radiation dose to the area of interest without irradiating the surrounding healthy organs and for a homogeneous dose distribution, as mentioned before. The thickness of the bolus depended on the required dose and the treatment technique.

**Figure 2:**
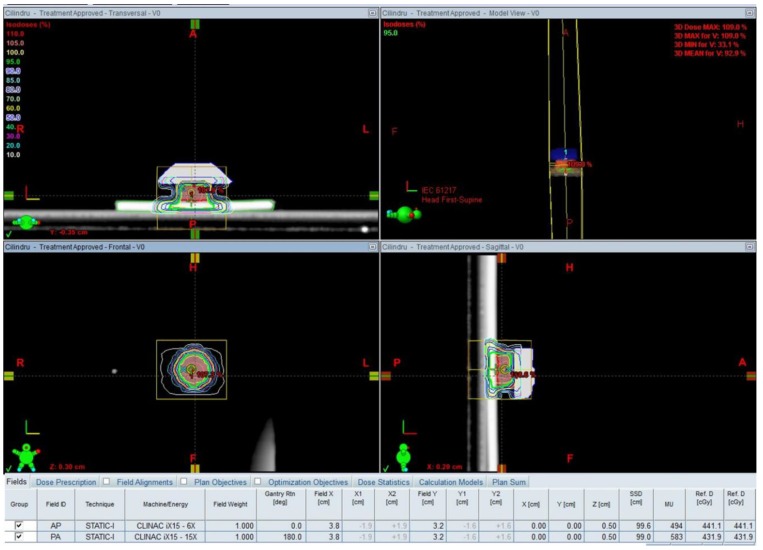
Parameters of the irradiation plan.

The parameters set for the radiation plan were: Two opposite, isocentric, equally weighted beams, one from the zero position of the Gantry (AP) with 6 MV and a posterior one positioned at 180 degrees (PA) with 15 MV (Figure 2).

### 2.4. Drug Loading

Before being incorporated into the scaffolds, the particles were loaded with doxorubicin in a colloidal suspension of 1% MNPs in water; 0.125% DOX was solubilized.

### 2.5. Characterization of the Magnetic Scaffolds

The chemical structure of all the prepared scaffolds was investigated by Fourier Transform Infrared Spectroscopy (FTIR) and the morphology by Scanning Electron Microscopy (SEM). Energy-dispersive X-ray spectroscopy (EDX) analysis has been performed to investigate the chemical composition of magnetic scaffolds. The scaffolds S1, S1R, S2, and S2R were analyzed and the interaction with phosphate buffer solution (PBS retention degree) was performed in in vitro degradation studies using an enzymatic complex of lysozyme (1200 μg/mL) and collagenase (100 μg/mL) [22]. Magnetic susceptibility for the scaffolds S1 and S2 was determined using magnetic susceptibility balance (MSB) Auto (Sherwood Scientific Ltd, Cambridge, UK). The reading of volume susceptibility, χV, or mass susceptibility, χm, displayed by the balance is proportional to the sample’s volume (or mass) present in the measuring region of the balance.

#### 2.5.1. In Vitro Drug Release

In vitro drug release was studied for the scaffolds S1-DOX (8 µg drug/mg scaffold) and S2-DOX (8 µg drug/mg scaffold) by immersing 20 mg of each sample in PBS, pH = 7.2, 0.01 M, into a dialysis bag that was completely immersed in another volume of PBS. The release study was performed at 37 °C for 14 days. A volume of the PBS was regularly removed for UV–Vis analysis and replaced with the same volume of fresh PBS. The absorbance was analyzed using the NanoDrop ND 1000 spectrophotometer (Thermo Fisher Scientific, Waltham, MA, USA) at 480 nm. A standard curve was used to assess the amount of DOX released.

#### 2.5.2. In Vitro Interaction Studies of Scaffolds with Cells

The interaction of all the prepared scaffolds with MG-63 (osteosarcoma) cells was studied. After adequate sterilization of the scaffolds (1 h exposure to UV radiation) and the preparation of a stock solution of DOX in sterile conditions, the scaffold aliquots and DOX were placed in direct contact with MG-63 bone cells. Cell viability was calculated using the MTT assay protocol [25].

## 3. Results

**Figure 3:**
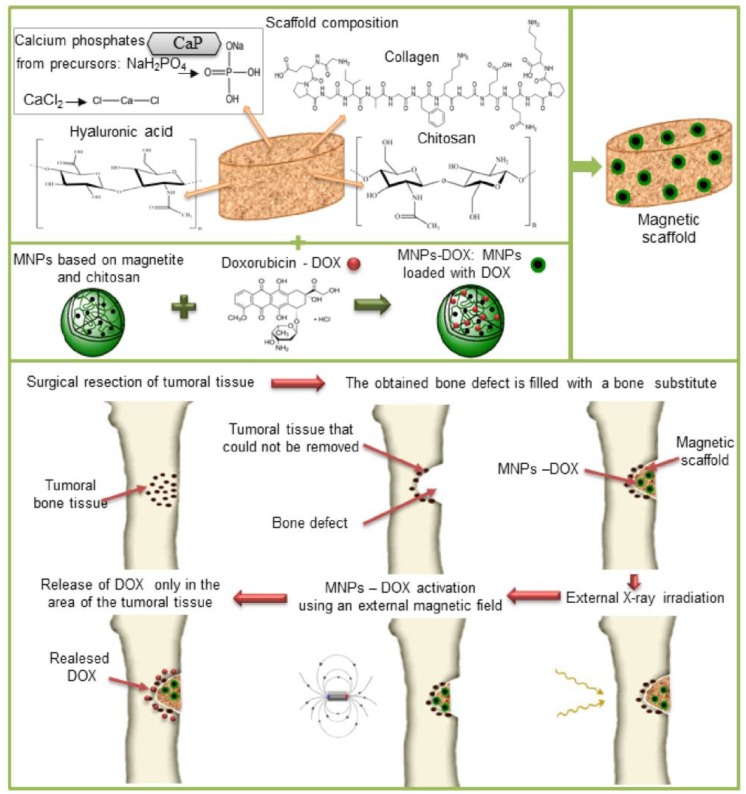
Treatment of bone tumors using radiotherapy and targeted chemotherapy.

Magnetic composite scaffolds based on biopolymers and calcium phosphates were obtained by the co-precipitation method and tested for combined therapy of cancer, which involves the use of radiotherapy in a first step and then the use of targeted chemotherapy, as schematically represented in Figure 3.

The X-ray irradiation of the scaffolds was achieved by performing some simple steps. First CT simulation was performed under similar conditions to irradiation used in clinical radiotherapy, including the laser positioning and the placement of radiopaque external markers, followed by scanning the irradiated volume with a dedicated CT scanner. The scanned CT images were used to draw the target volume. Then, the radiation beam parameters were determined (working energy, incident angles, dimensions and shapes, weighting, accessories). Finally, the technique and parameters previously set were transmitted and implemented to the particle accelerator.

**Figure 4:**
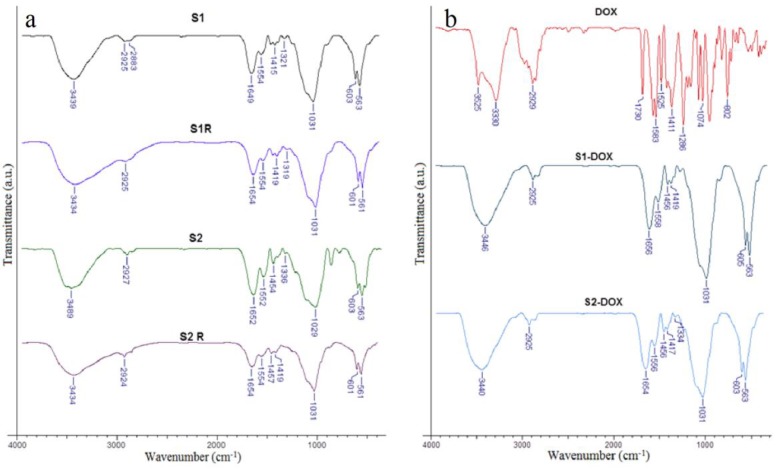
The chemical structure of the scaffolds. (**a**) Normal scaffolds (S1, S2) and irradiated scaffolds (S1R, S2R); (**b**) DOX and S1-DOX, S2-DOX scaffolds.

It is very important to note that the X-ray dose did not modify the chemical structure of the scaffolds. Specific peaks for the three biopolymers, calcium phosphates, and MNPs can be observed in all FTIR spectra of magnetic scaffolds (Figure 4a). The FTIR spectra of the scaffolds containing MNPs loaded with DOX and the spectra of DOX are shown in Figure 4b.

**Figure 5:**
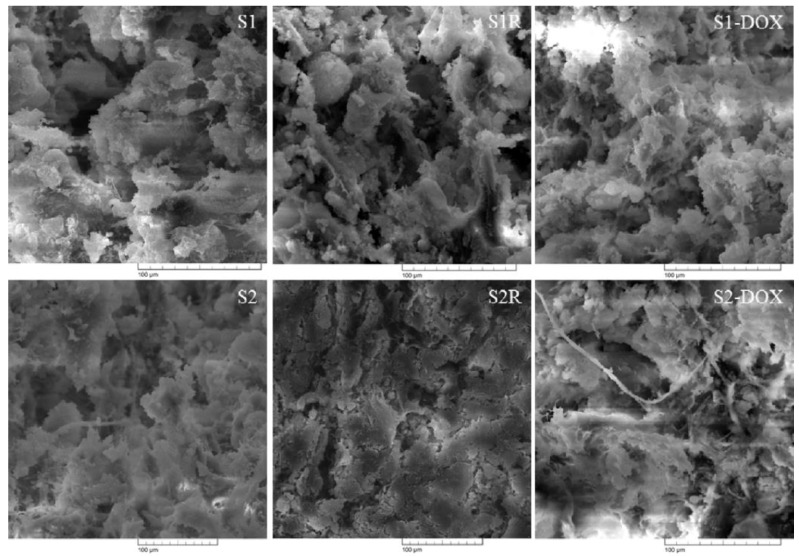
The morphology of the scaffolds.

The morphology of the scaffolds intended for bone regeneration is a very important feature to take into consideration, therefore, it was investigated for both irradiated and normal scaffolds, the results being displayed in Figure 5.

Uniform distribution of the MNPs and calcium phosphates in the polymeric phase was observed. The homogenous distribution of the magnetic particles is due to their direct incorporation into synthesis; similar data has been found in other studies [26]. Similar porous morphologies have been observed for both irradiated and non-irradiated scaffolds.

The interactions of the scaffolds with PBS were monitored using a volumetric method and the maximum retention degree of the scaffolds after 72 h was calculated. The values obtained, detailed in Table 1 (a minimum of 790% for S1R and maximum of 1040% for S2), are due to the porous structure of the scaffolds and their biopolymeric composition.

**Figure 6:**
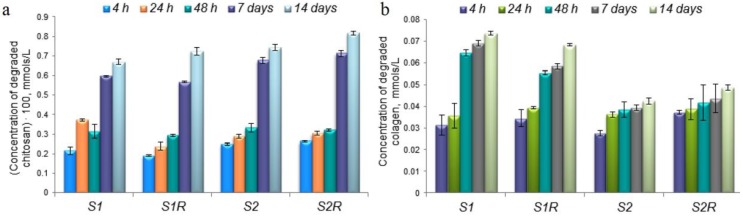
In vitro degradation of the scaffold: (**a**) Degraded chitosan; (**b**) degraded collagen.

Enzymatic degradation of the scaffolds was studied using a complex of two specific enzymes, lysozyme and collagenase, and the results are shown in Figure 6. A gradual increase can be observed in the concentration of a degraded polymer, chitosan (Figure 6a) or collagen (Figure 6b).

**Figure 7:**
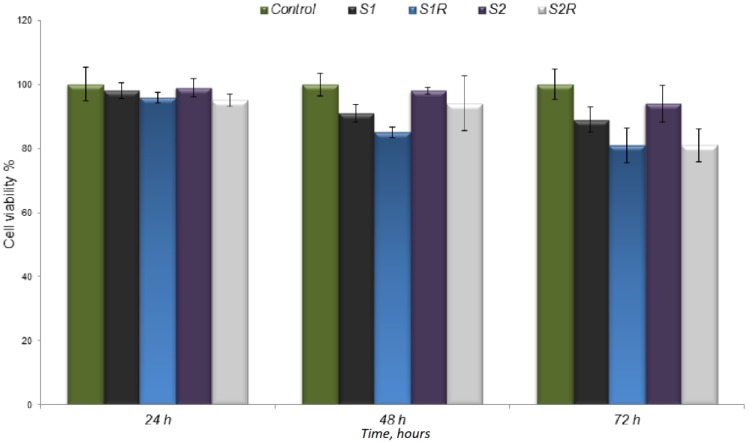
The cell viability measured by MTT assay.

The interaction of the irradiated and non-irradiated scaffolds with cells was analyzed by direct contact with osteoblasts (Figure 7). The cell viability value decreased over time; at 72 h there was a difference of about 10 percent between the non-irradiated and the irradiated scaffolds.

The results of the first part of the study showed encouraging results, namely the chemical structure of the scaffolds, their morphology, and the enzymatic degradation behavior were not influenced by the X-rays used in radiotherapy, meaning that the scaffolds can be further used in chemotherapy.

The second step was to load Doxorubicin, a chemotherapeutic agent, in the MNP structure and then incorporate the MNPs-DOX into the scaffold.

**Figure 8:**
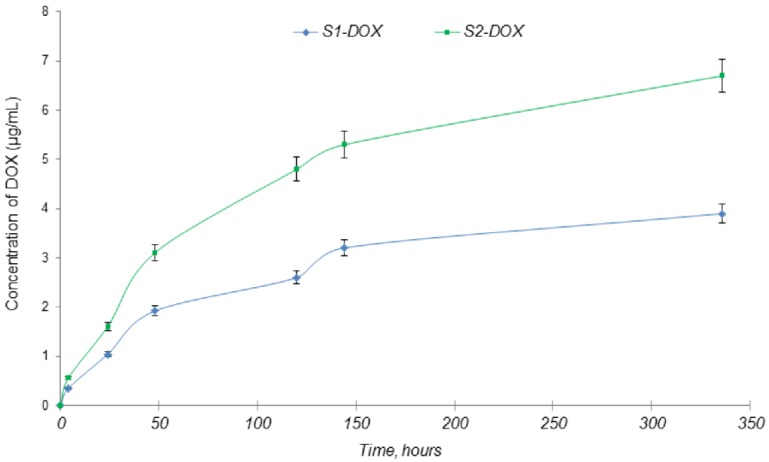
In vitro drug release of DOX from scaffolds.

Regarding the in vitro DOX release from the scaffolds, a gradual release can be observed in Figure 8. For the S1-DOX scaffold, the drug release was more constant over time, probably due to the fact that this scaffold has a considerable amount of chitosan in its composition compared with S2-DOX and the drug strongly bonded to the polymer.

**Figure 9:**
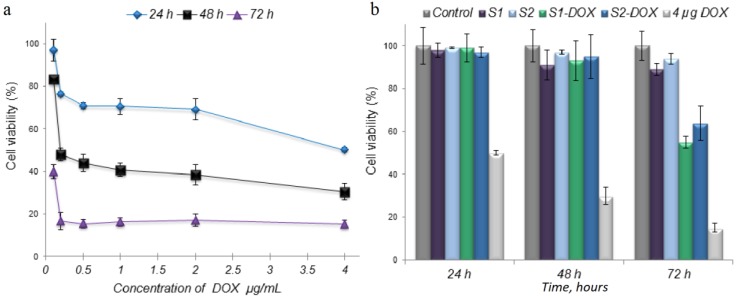
Interaction of the drug and scaffolds with cells. (**a**) Interaction of DOX with cells; (**b**) Interaction of scaffolds containing MNPs loaded with DOX with cells.

In vitro interaction of the scaffolds with cells was analyzed using the MG-63 cell. The viability of the cells in direct contact with all the scaffolds was between 97% and 99% for the first two contact times, 24 and 48 h (Figure 9b). For normal scaffolds, the values decreased to 89% in the case of S1 and to 94% in the case of S2. However, there was a significant decrease in the viability of osteoblasts at 72 h of contact for the scaffolds containing MNPs-DOX. The values obtained were 55% for S1-DOX and 64% for S2-DOX. The anti-tumor effect of DOX on the cells is shown in Figure 9a.

## 4. Discussion

In the case of some bone tumors, the first step is to surgically remove the tumor. After tumor resection, a bone substitute is used to fill the resulting bone defect followed by treatment involving radiotherapy and/or chemotherapy. Scaffolds that mimic bone structure with the inclusion of MNPs, named magnetic scaffolds, may be used as a bone substitute.

Because radiotherapy is first used in clinical practice, it is important to prove that X-rays do not influence the scaffolds’ properties. Chemotherapy may also be necessary and due to its side effects [27], targeted drug delivery could be a promising solution. For this purpose, the MNPs included in the scaffolds were loaded with chemotherapeutic drugs.

In order to analyze the influence of X-rays on magnetic scaffolds, a radiation dose of 8 Gy/single fraction was used. This dose is commonly administered in bone metastases irradiation [28,29,30].

The chemical structure of the scaffolds was studied using FTIR. Regarding the biopolymers, the following characteristic peaks can be noted: 3435 cm^−1^, 3431 cm^−1^, and 3434 cm^−1^ for the hydroxyl group; 2925 cm^−1^ for –CH_2_; 1656 cm^−1^ and 1654 cm^−1^ for amide I; 1556 cm^−1^ and 1558 cm^−1^ for amide II; and 1237 cm^−1^ and 1239 cm^−1^ for amide III [31]. For calcium phosphates, there were observed peaks of phosphate bands at 601 cm^−1^ and 602 cm^−1^ and representative bands for MNPs at 561 cm^−1^ were noted [21,32].

Concerning the DOX spectra (Figure 4b), the following characteristic bands are important to mention the succeeding characteristic bands: 2929 cm^−1^ for the stretching vibration of C–H; 1730 cm^−1^ for the stretching vibration of C=O; 1617 cm^−1^ and 1525 cm^−1^ for the bending vibrations of N–H; 1411 cm^−1^ for the stretching vibration of C–C; and 1286 cm^−1^ for the stretching vibration of C–O–C. Some of these bands can be seen in the spectra of the scaffolds S1-DOX and S2-DOX (Figure 4b), suggesting the interaction of DOX with the scaffold components [33]. Also, on this spectrum the following peaks characteristic for biopolymers are present: 3446 cm^−1^ and 3440 cm^−1^ for the hydroxyl group –OH; 2925 cm^−1^ for –CH2; 1654 cm^−1^ and 1656 cm^−1^ for amide I; and peaks with values close to those of the scaffolds without DOX (Figure 4a).

In terms of scaffold morphology, the same porous structure was observed for all the scaffolds and also the integration of MNPs/MNPs-DOX and calcium phosphate in the polymeric matrix. Porosity is a specific feature of scaffolds, with applications in the field of bone tissue engineering. The pore size of the scaffolds must be taken into account. Open porous and interconnected networks strongly influence cell nutrition, proliferation, and migration for tissue vascularization. Porous structures allow an efficient release of biofactors, such as bioproteins, genes, drugs, or cells [34].

PBS is a buffer very often used for testing various scaffolds’ interaction with biological fluids because it can provide some information regarding the hydrophilicity and swelling of materials, as well as its disintegration in an aqueous medium. The fluid retention also influences the release of the drug from the scaffold because the aqueous medium is a vehicle for bioactive diffusion in surrounding environments.

According to Brouwer et al. [35], the concentration of lysozyme in human serum is 950–2450 μg/L, but increased levels can be observed in benign diseases like inflammatory bowel disease, many blood disorders like polycythemia vera, multiple myeloma, and malignant processes like leukemia [36]. For example, Firkin [37] reported a very high serum lysozyme level of 30–120 μg/mL in chronic myeloid leukemia and myelofibrosis. Concerning these data, we chose to use a concentration of 1200 μg/mL, considering the fact that our scaffolds are intended for bone tumor treatment and an elevated level of lysozyme will be found in the bone.

Because collagen is the most predominant protein in the human body, collagenase levels are difficult to measure with precision, this enzyme being found in all tissues and organs where collagen exists. Interstitial collagenase has a key role in normal and pathological remodeling of collagenous extracellular matrices, including skeletal tissues [38]. Also, there are different types of collagenase, e.g., in human skin, fibroblasts secrete collagenase as two proenzyme forms (57 and 52 kDa) [39].

The rate of drug release from scaffolds is dependent on their degradation rate. Also, the MNPs included in the scaffolds are coated with chitosan and therefore lysozyme, the enzyme involved in the chitosan degradation process, will also contribute to the drug release.

The obtained values for the in vitro degradation study and for the interaction with PBS and the scaffolds exposed to X-rays versus the simple ones are comparable, confirming that X-ray radiation did not affected the scaffolds’ composition.

On the other hand, the result obtained for the interaction of the scaffolds with cells should not be interpreted as negative, as the role of radiation is to destroy the MG-63 tumor cells. The underlying phenomenon is difficult to understand and complex studies are needed to reveal the mechanism.

Doxorubicin (DOX) is a potential drug for the treatment of bone tumors and its use is limited by its systemic side effects. It was used for the second part of the study. DOX is an anthracycline antibiotic, which, at the molecular level, interacts with DNA and interferes with nucleic acid synthesis, resulting in a remarkable effect on DNA transcription [40,41].

For bone chemotherapy applications, DOX is used in drug delivery systems manufactured from different magnetic scaffolds/composites: Nanocrystalline apatite [20], hydroxyapatite [42], chitosan and hydroxyapatite [43], poly lactic acid-co-glycolic acid–polyethylene glycol (PEG–PLGA) [18], and gelatin magnetic microspheres [44].

The sudden decrease in viability of the cells that had direct contact with the scaffolds S1-DOX and S2-DOX could be explained by the fact that DOX is gradually released from the scaffolds at a relatively low ratio. This slow release property of the scaffolds could likewise be taken into consideration for other strong anti-tumor drugs or drug combinations that could be transported to the target site and released at an optimal ratio for tumor control [45].

## 5. Conclusions

This paper’s purpose was to study the influence of X-rays on composite scaffolds based on biopolymers, calcium phosphates, and MNPs and to evaluate them as drug delivery systems for radio-chemotherapy. An X-ray radiation dose, similar to the one prescribed for bone metastases irradiation, did not influenced the scaffold features, like structure, composition, morphology, in vitro degradation properties, and interaction with cells. The scaffolds containing MNPs loaded with doxorubicin exhibited a gradual and slow release of the drug. These characteristics are good premises for future experiments aiming to confirm the suitability of magnetic scaffolds for combined therapy of malignant bone tumors.

## Figures and Tables

**Table 1:** Magnetic scaffold composition and distinctive features.

Magnetic Scaffold	Biopolymer Composition	Ca/P (Initial)	Ca/P (Final)	MNPs Concentration	PBS RD%	Magnetization (emu/g)	Distinctive Features
S1	28.79% Col,	1.65	1.63	5% MNPs	995 ± 30	22.41 ± 1.41	Used as control
	71.21% Cs						
S2	50% Cs,	1.65	1.64	5% MNPs	1040 ± 35	44.42 ± 0.92	Used as control
	50% Col						
S1R	28.79% Col,	1.65	1.63	5% MNPs	990 ± 50	n.a.	X-ray irradiated
	71.21% Cs						
S2R	50% Cs,	1.65	1.64	5% MNPs	987 ± 19	n.a.	X-ray irradiated
	50% Col						
S1-DOX	28.79% Col,	1.65	1.62	5% MNPs-DOX	n.a.	n.a.	MNPs loaded with DOX
	71.21% Cs						
S2-DOX	50% Cs,	1.65	1.63	5% MNPs-DOX	n.a.	n.a.	MNPs loaded with DOX
	50% Col						

Col—collagen, Cs—chitosan, MNPs—magnetic nanoparticles, PBS—phosphate buffered solution, RD—retention degree, DOX—doxorubicin.
